# Retrorectal Epidermal Inclusion Cyst: An Incidental Finding During Cesarean Section

**DOI:** 10.7759/cureus.11300

**Published:** 2020-11-02

**Authors:** Sundus Nasim, Sohail Kumar, Dua Azim, Lajpat Rai, Summaya Saeed

**Affiliations:** 1 Internal Medicine, Dow Medical College, Dr. Ruth K. M. Pfau Civil Hospital, Karachi, PAK; 2 Surgery, Dow Medical College, Dr. Ruth K. M. Pfau Civil Hospital, Karachi, PAK

**Keywords:** retrorectal, presacral, epidermoid, cyst, inclusion cyst

## Abstract

An epidermal inclusion cyst is a benign lesion, frequently observed throughout the body. However, its presence in the retrorectal region is a rare occurrence. With a higher incidence in women and non-specific symptoms, these cysts are mostly an incidental finding. Here, we report a case of a 33-year-old female presenting with complaints of abdominal pain and sensation of incomplete and painful defecation. The patient’s history revealed that a large pelvic cyst was found incidentally during her first cesarean section (CS) owing to an arrest in labor. A presumptive diagnosis of rectal duplication cyst was made based on digital rectal exam (DRE), computed tomography (CT), and magnetic resonance imaging (MRI) findings. For cyst removal, the patient underwent a midline laparotomy. A postoperative biopsy led to the confirmed diagnosis of the retrorectal epidermal inclusion cyst. On follow-up, our patient showed a smooth recovery without any complications. We conclude that when dealing with a pregnant woman with a pelvic mass, developmental cysts should always be kept in mind. Timely diagnosis and management of a retrorectal mass is essential for effective treatment and to prevent subsequent complications.

## Introduction

The retrorectal or presacral space is a clinically significant region that harbors many pathologies. An epidermal inclusion cyst, also known as an epidermoid cyst, is an infrequent entity among the cystic pathologies found in the retrorectal region [1]. An epidermal inclusion cyst is a benign congenital lesion that develops from the remnants of an ectodermal tissue misplaced during embryogenesis. It typically appears in women of reproductive age [2]. It can occur at various locations in the human body; the most frequent sites include the scalp, face, neck, and trunk. The presence of these cysts in the presacral area, however, is relatively rare, which makes this an interesting case to report. Evidence suggests that epidermal cysts account for approximately 6% of all the presacral tumors [3]. They commonly develop from areas of previous surgery or trauma [4], which may result in the epidermis to sequester into the dermis. The lesions are susceptible to infection, development into a fistula, or malignant transformation. Compression of surrounding structures such as the rectum, development of symptoms (constipation, lower abdominal pain, and dysuria), and features hinting a malignant transformation are indications of surgery, which remains the best treatment option [5].

Here, we report a case of a retrorectal epidermal inclusion cyst in a 33-year-old female who presented to us with a chronic history of abdominal pain and painful defecation. This report also highlights the importance of imagining techniques in diagnosis and the use of midline laparotomy in the successful treatment of the patient.

## Case presentation

A 33-year-old female presented to the outpatient department with complaints of lower abdominal pain and painful defecation for the last two months. She also reported a sensation of incomplete defecation for five years. Past medical history was insignificant for any constitutional symptoms or rectal bleed. Moreover, surgical history revealed that the patient delivered her first child seven years ago via cesarean section (CS) due to the arrest of labor; at that time, an intrapelvic cyst was detected during the operation. The patient remained asymptomatic and did not seek any treatment for the cyst. The delivery of the second child via CS three years ago was uneventful. No pathology, aside from the previously noted cyst, was identified during the second CS.

On examination, there was a scar of CS on inspection; the abdomen was soft and non-tender. No mass was palpable on examination. The vaginal examination revealed a bulge in the posterior fornix that was suggestive of a rectal growth. Digital rectal examination (DRE) showed an extra-mural bulge, 3 cm from the anal verge. Colonoscopy findings and the remainder of the systemic examinations were unremarkable.

**Figure 1:**
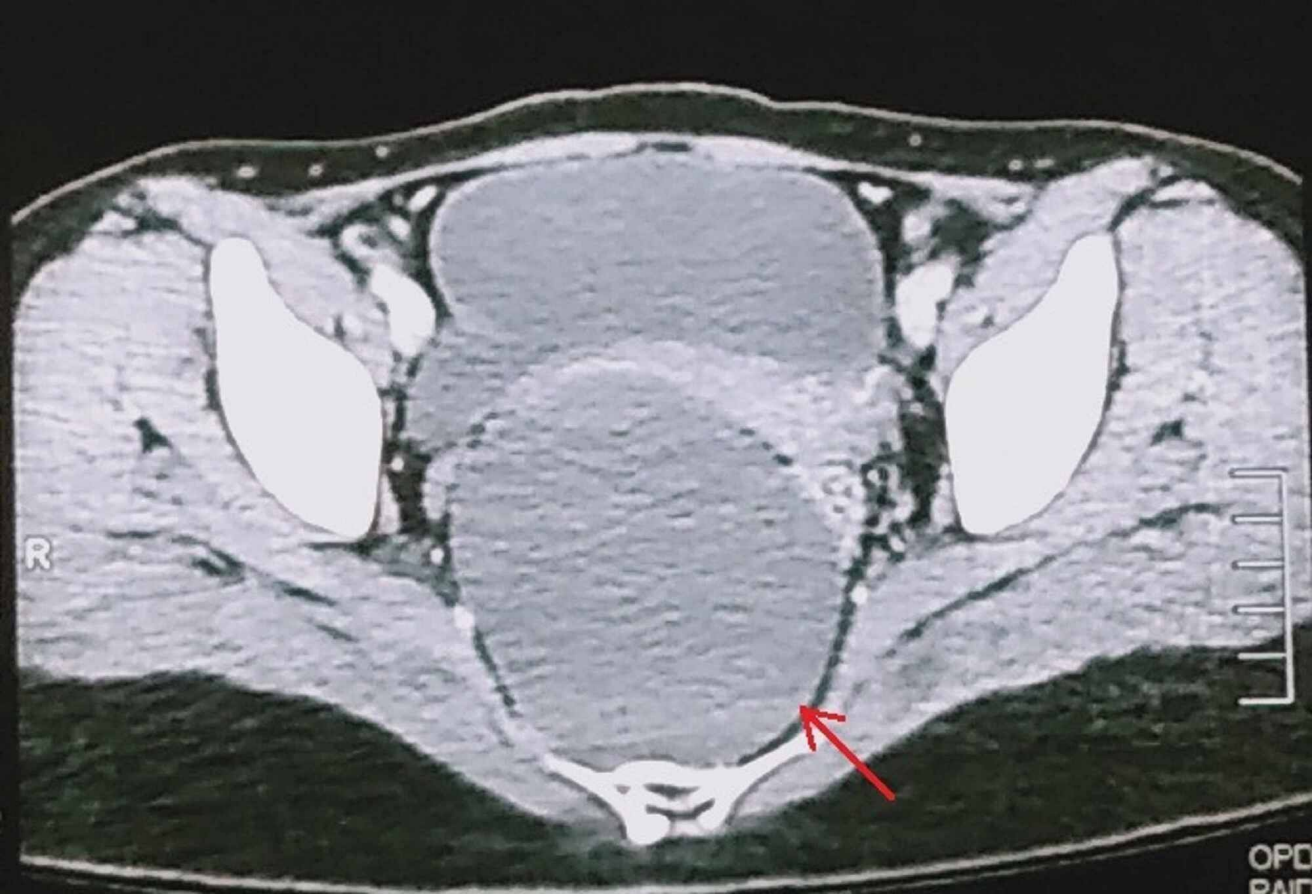
CT scan showing a well-defined cystic area anterior to the sacrum, displacing the rectum towards the left CT, computed tomography

**Figure 2:**
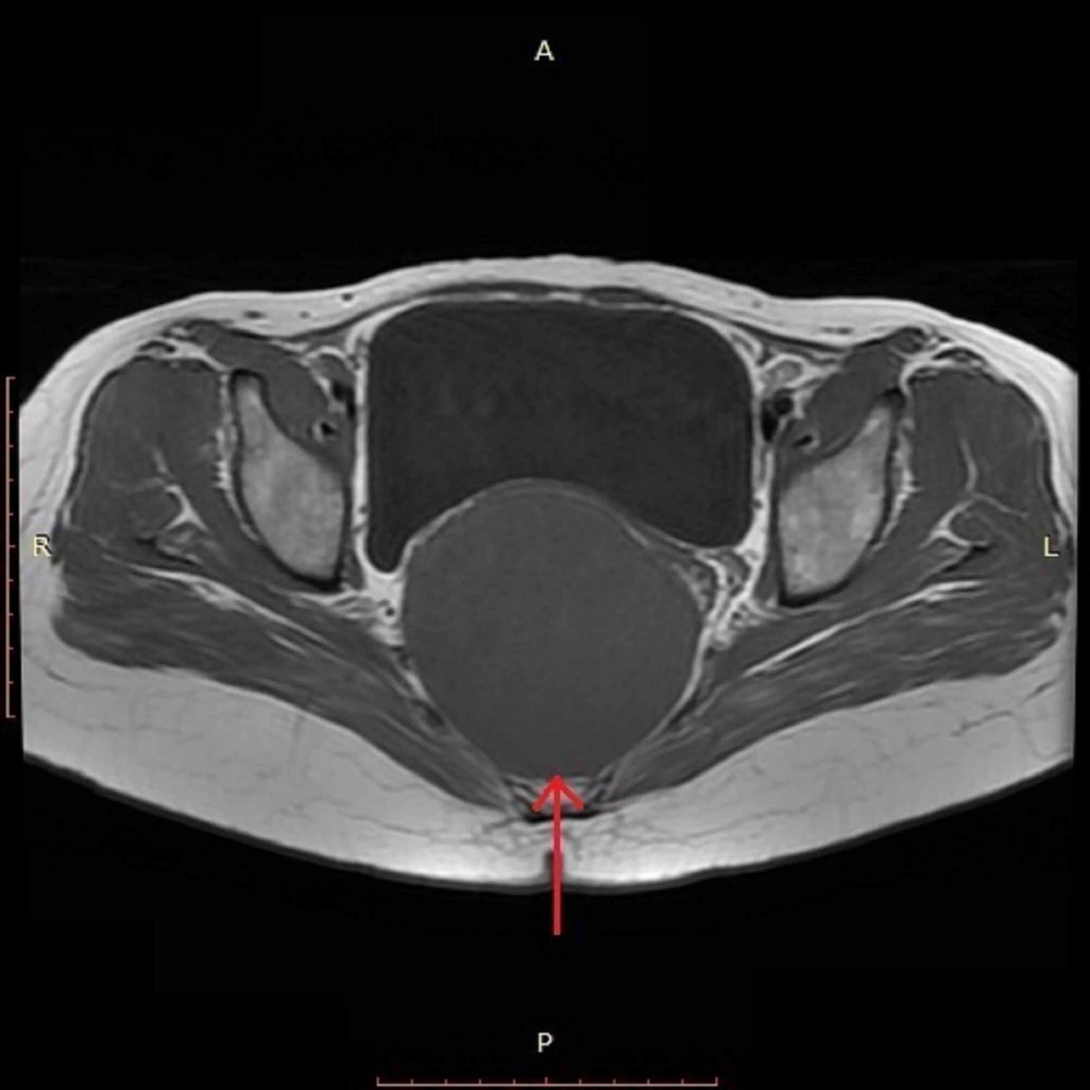
T1W-MRI showing a large, hypointense lesion in the presacral space T1W, T1-weighted; MRI, magnetic resonance imaging

**Figure 3:**
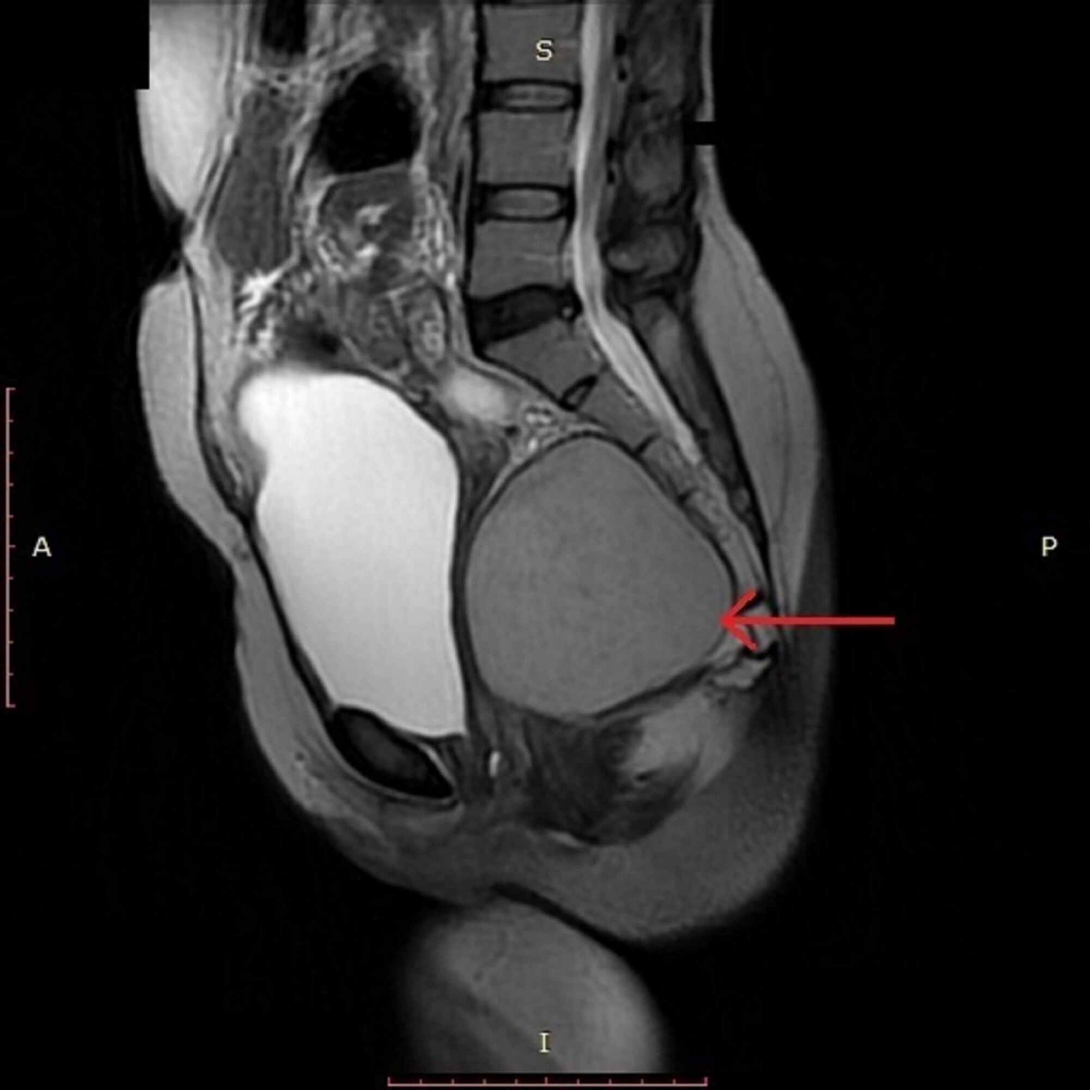
T2W-MRI showing a large, slightly hyperintense lesion in the presacral space T2W, T2-weighted; MRI, magnetic resonance imaging

Contrast-enhanced computed tomography (CT) scan of the pelvis was performed, which revealed a large, well-defined cystic area anterior to the sacrum measuring approximately 8.8 x 8.7 x 8.3 cm (Figure 1). This cyst was displacing the rectum towards the left side and the lower uterine segment and urinary bladder anteriorly, thus resulting in significant compression over these viscera. Subsequently, contrast-enhanced pelvic magnetic resonance imaging (MRI) was done to further characterize the lesion. MRI revealed a large, abnormal signal intensity lesion (8.8 x 8.4 x 8.2 cm) in the pelvis extending up to the presacral space, appearing hypointense on T1-weighted (T1W) and slightly hyperintense on T2-weighted (T2W) images, indicating peripheral post-contrast enhancement without signal suppression short-T1 inversion recovery (STIR) images (Figure 2, 3). No solid enhancing component was seen. The lesion was found to be inseparable from the rectum, which was displaced towards the left anterolateral aspect. There appeared to be no continuity of the lesion with the surrounding structures. These imaging findings were consistent with the presumed diagnosis of presacral/rectal duplication cyst. 

**Figure 4:**
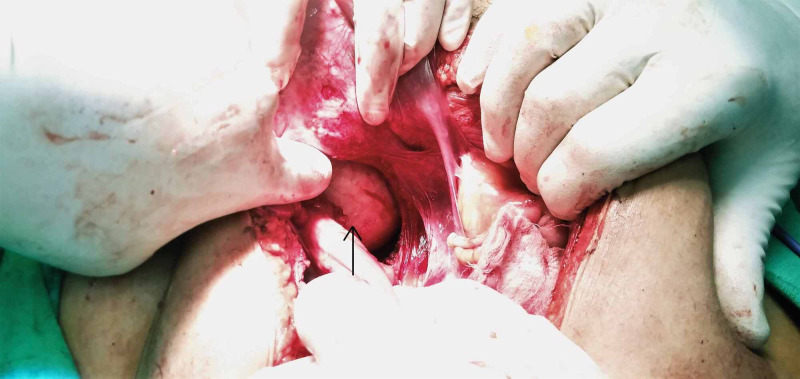
Intraoperative image showing a large retrorectal cyst in the presacral space

**Figure 5:**
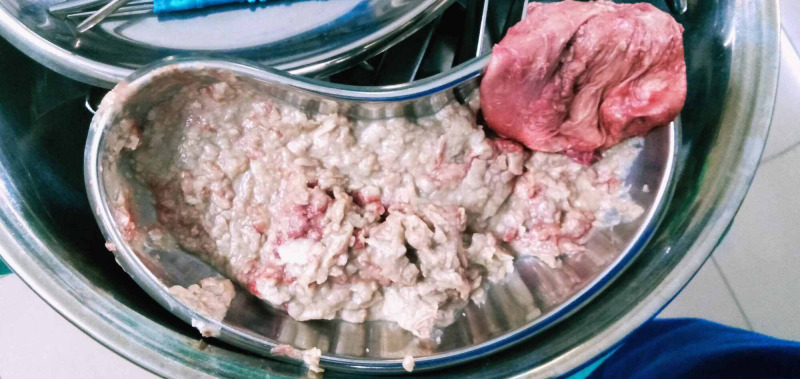
Large amounts of cheesy material released due to rupture of the cyst during excision

The patient then underwent laparotomy via a lower midline incision, which revealed a cyst on the right side in the presacral space, firmly adherent to the sacrum (Figure 4). Thus, we performed a total surgical excision of the cyst and preserved the adjacent structures. However, the cyst ruptured during excision, which released copious amounts of cheesy material (Figure 5). Histopathological reports revealed a benign cystic lesion with focal metaplasia of the lining epithelium. Thus, a definitive diagnosis of retrorectal epidermal inclusion cyst was made. The symptoms resolved following cyst removal. The patient remained asymptomatic during a one-year follow-up period and showed no sign of recurrence of the disease.

## Discussion

The retrorectal space, also known as the presacral space, is a potential space that lies anterior to the sacrum and coccyx and posterior to the rectum. Retrorectal tumors are uncommon and comprise a heterogeneous group of tumors that are mostly benign; however, malignant tumors are also seen occasionally. Owing to their rarity, the exact incidence of retrorectal tumors is unknown. These lesions are broadly categorized into congenital, mesenchymal, osseous, neurogenic, and miscellaneous. Moreover, they are further sub-grouped into benign or malignant as well as cystic or solid [3,6]. Congenital lesions are relatively common, accounting for more than 50% of all retrorectal tumors. Approximately two-thirds of congenital masses are developmental cysts (epidermoid, dermoid, enteric, tailgut, and duplication cysts) [1].

Retrorectal epidermoid cysts are congenital lesions originating from the ectoderm and are typically present in women of reproductive age [2]. While epidermal inclusion cysts can occur in various regions throughout the body, especially on the trunk, back, scalp, and face, they are very rarely seen in the retrorectal location. Although many theories have been put forward concerning the etiopathogenesis of epidermal cysts, the exact cause remains unknown. Studies suggest they commonly arise spontaneously, but they may also arise secondary to traumatic surgery resulting in implantation of the epithelium into the sub-epithelial tissue [4,7]. However, before the incidental finding of the pelvic cyst, the past surgical history of our patient was unremarkable.

Detailed clinical evaluation, including medical history and proper physical examination, is essential for establishing the correct diagnosis [8]. Most of the affected individuals are asymptomatic, and the cyst is discovered incidentally either on imaging or per-operatively for various reasons, as was seen in our case. Symptoms, if present, are non-specific and arise from the compression of surrounding organs. Patients may experience lower abdominal pain, constipation, and incomplete defecation due to pressure over the rectum. Presence of dysuria and increased urinary frequency suggests the involvement of the lower urinary tract [6]. Our patient displayed symptoms that indicated compression over the rectum without urinary system involvement. Thus, owing to its non-specific clinical presentation and diverse pathology, the diagnosis is often delayed. Studies have also reported that retrorectal cyst may obstruct pelvic outlet during labor, necessitating CS, as described in our case [9]. In our case, the retrorectal cyst presented as an extramural palpable mass posterior to the rectum on DRE, which is consistent with the previous findings. As per clinical evidence reported by Lev-Chelouche et al., 100% of the patients under consideration presented with a palpable retrorectal mass on DRE, making it the least expensive and most effective method of prompt diagnosis of a presacral tumor [10]. Laboratory tests have no diagnostic value for retrorectal cysts [7].

According to a literature review, several imaging modalities are available to assess and diagnose retrorectal cysts, including CT, MRI, ultrasonography, endoscopy, and barium enema. CT of epidermoid cyst reveals discrete, homogenous, thin-walled lesions with fluid density. CT also provides evidence of anterior rectal displacement along with the absence of associated calcifications. Moreover, on MRI, T1W images of epidermoid cysts demonstrate low-intensity signals, whereas T2W images show lesions with high-intensity signals. Nevertheless, a preoperative biopsy is discouraged due to the risk of possible infection and potential seeding of malignant cells to other regions [11]. Imaging techniques that we used to establish a preoperative differential diagnosis in our patient were CT, MRI, endorectal ultrasound, and colposcopy. These modalities not only strengthened our presumed diagnosis of a presacral cyst, but also allowed us to accurately assess the size of the lesion, its cystic nature, and its relationship to the adjacent structures. However, our corroborative diagnosis was made based on intraoperative and postoperative histopathological findings.

Complete surgical removal of epidermal cysts and other retrorectal tumors is the only treatment option. Surgery is indicated when signs and symptoms are suspicious of either compression of the surrounding structures and malignant transformation or to prevent common complications such as infections and fistula formation [5,12]. Moreover, surgical resection is also recommended to establish a definitive diagnosis, as evident in our case. Traditionally, these cysts are excised through anterior transabdominal (laparotomy) or transperineal approaches. In recent years, minimally invasive techniques, such as laparoscopic and robotics surgery, have been safely used [12]. These are associated with enhanced recovery, improved cosmetic outcomes, decreased morbidity, and reduced postoperative pain. However, minimally invasive approaches are avoided in the case of a narrow pelvis or large masses [12]. Thus, the anterior transabdominal approach is preferred if the lesion is greater than 5 cm and the lower extent of the lesion is not below the fourth sacral level [8]. We used a transabdominal approach to surgically resect the unusually large cyst with the greatest diameter of 8 cm.

Keeping in mind the rarity of retrorectal masses, we recommend that if an intrapelvic mass is observed for the first time in pregnancy, the risk of retrorectal epidermal cysts should be kept in mind. Complete surgical excision of the cyst is the only definitive treatment and should be performed as early as possible due to the risk of developing complications such as compression symptoms, infection, and malignant transformation. However, any surgical procedure during pregnancy may pose a risk of fetal death and premature delivery. Our goal, therefore, is to emphasize the approach that prompt diagnosis of retrorectal cysts in a pregnant woman, followed by the timely decision of a suitable mode of delivery and the management of the cyst, is essential to avoid further complications. To date, there are no suggested criteria regarding the definitive mode of delivery in such cases. More work is required to develop preoperative diagnostic and management strategies in the care of a pregnant woman with retrorectal mass.

## Conclusions

The rare incidence of an epidermal inclusion cyst in the retrorectal space requires early diagnosis and complete surgical excision. This is essential to decrease the risk of recurrence and possible complications. Moreover, in a pregnant woman with a retrorectal cyst, the likelihood of difficult labor increases. Thus, using various imaging techniques, an accurate diagnosis and therapeutic approach are advised. Meanwhile, more research is required to determine the exact etiology and pathogenesis of these cysts.
